# Bay Leaves Extracts as Active Additive for Food Protective Coatings

**DOI:** 10.3390/foods12203741

**Published:** 2023-10-11

**Authors:** Manuel Peña-Ortiz, Luis Serrano, Antonio A. Romero, Araceli García

**Affiliations:** 1 FQM-383 NANOVAL Group, Organic Chemistry Department, University of Córdoba, Campus de Rabanales, Marie Curie Building, Ctra. Nnal. IV-A, Km 396, 14014 Córdoba, Spain; b52penom@uco.es (M.P.-O.); qo1rorea@uco.es (A.A.R.); 2 BioPrEn Group (RNM 940), Chemical Engineering Department, Instituto Químico para la Energía y el Medioambiente (IQUEMA), Faculty of Science, University of Córdoba, 14014 Córdoba, Spain

**Keywords:** *Laurus nobilis*, strawberry, edible coating, antioxidant, antimicrobial

## Abstract

Ethanolic extracts of bay leaves were obtained using the Soxhlet method (extraction yield of 22.3 ± 1.2%) and further analyzed through different methods, thus determining the chemical composition with gas chromatography, phenolic content with the Folin–Ciocalteu technique (11.8 ± 0.4% wt.) and antioxidant power with the radical 2,2′-Azino-bis(3-ethylbenzothiazoline-6-sulfonic acid) method (75.06%). Furthermore, its effect on the growth of two bacteria, *Escherichia coli* and *Bacillus cereus*, and on two yeasts, *Candida glabrata* and *Saccharomyces cerevisiae*, was determined, showing a minimum inhibitory concentration of 0.65 mg/mL on the growth of *B. cereus*. Finally, edible films were prepared using different polymers (carboxymethyl cellulose, gum Arabic, polyvinyl pyrrolidone, and polyvinyl alcohol) containing 0, 5, 10, or 15% wt. of bay leaf extract as troubleshooting for perishable fruits, specifically for cultivated strawberry. The prepared composites presented reduced water vapor permeabilities (up to 4.3 × 10^−7^ g·Pa^−1^·m^−1^·h^−1^), high specific transparencies (≈30%/mm), as well as the effective blocking of ultraviolet radiation (>99.9%). In vivo tests showed that the most suitable treatment for strawberry protection was the impregnation with a composite comprising polyvinyl alcohol and a 15% wt. bay leaf extract, resulting in a noteworthy reduction in mass loss (22% after 6 days). It can be asserted that food packaging with the designed composites would be an effective alternative for the reduction in postharvest losses.

## 1. Introduction

According to United Nations projections, the world’s population could rise to 9.15 billion by 2050, meaning that the supply of food products will only be possible by increasing their production by more than 70% [1]. The development of molecular biology and the intensive use of fertilizers are currently seeking to meet this objective, although new approaches to minimizing postharvest losses through affordable packaging procedures are also being studied.

Fruits, one of the most perishable food groups, require proper handling since harvesting and subsequent refrigeration, which is directly associated with the problem of energy expenditure during transport and storage [2]. One of the perishable fruits par excellence, *Fragaria × ananassa* (commonly known as cultivated strawberry), has a shelf life of 5–7 days to ensure its quality and safety. Being the most consumed berry globally for its multiple properties and benefits, as a non-climacteric fruit, the increase in the concentration of the abscisic acid phytohormone after its harvest, in addition to its high respiration rate, determines the generation of water loss, mechanical damage, and fungal growth, meaning this short postharvest life [3].

Against these drawbacks, research has explored multiple materials with the ability to significantly extend the shelf life of strawberries while being less harmful to the environment, including their packaging and coating by using bioplastics such as polyvinyl alcohol (PVA) and chitosan [4]. Edible coatings are wrappers that form a thin, semi-permeable barrier on the surface of foods. They serve multiple purposes, enhancing barrier properties (reducing transpiration, loss of water…), microbiological safety (minimizing the risk of contamination), and structural integrity (mitigating mechanical damage from punctures and handling during storage) [5,6]. Notably, transparent coatings that preserve the original color of food are often preferred, resulting in a glossy surface that enhances the visual appeal of the foodstuff [7].

To date, edible coatings have been based on different polymers (carboxymethyl cellulose, gum Arabic…), together with other additives. As for the latter, the growing interest in the valorization of agro-industrial byproducts (whey proteins, collagen, pomegranate peel…) and other natural substances, such as aloe vera leaves, organic acids, and vegetable essential oils (EOs), has prompted research into their utilization for this purpose, seeking to use their inherent bioactive properties to enhance the multifunctionality of the designed products [8,9].

EOs are a group of generally aromatic liquids extracted from different parts of plant species that have been widely used to improve the shelf life of food products due to their rich content of bioactive molecules. These extracts have generally been obtained through their affinity for some organic solvents. In the case of the Soxhlet method, the reflux of the liquid phase ensures the contact of fresh solvent with the solid sample, which has been used routinely for the extraction of EOs from plant biomass in multiple investigations [10].

Laurel (*Laurus nobilis*), also called bay leaf, is a small evergreen tree belonging to the Lauraceae family, native to the Mediterranean area. Its EO is mainly composed of tannins, flavonoids, alkaloids, anthocyanins, traces of fats, and many minerals, among others, which provide a strong and pleasant smell to the oil, as well as biological activities such as antioxidant, antimicrobial, insect repellent, healing, immunostimulant, and anti-inflammatory [11]. For all these reasons, laurel has been cultivated extensively.

Considering all the above, we set out to use the bay leaf EO as a natural additive in different polymeric matrices (i.e., carboxymethyl cellulose, gum Arabic, polyvinylpyrrolidone, and polyvinyl alcohol) as a method to extend the shelf life of cultivated strawberry by edible coating, trying to demonstrate the potential suitability of the designed treatment for strawberries as well as other traded perishable foods.

In terms of economic feasibility, the substantial byproducts produced by *L. nobilis* during pruning (over 90% of the annual laurel production is discarded) underscore the potential for their utilization within the framework of a biorefinery process that aligns with the principles of a circular economy [12]. Furthermore, multifaceted edible coatings help to maintain the physiological and physicochemical properties of food over an extended period of time, leading to a reduction in post-harvest losses. However, the choice of the materials comprising them is crucial, since it is estimated that raw material costs contribute to 70% of the total edible coating expenditure. Thus, the availability, extraction method, and cost of additives play a key role in this food protection approach [13].

## 2. Materials and Methods

### 2.1. Materials

Bay (*L. nobilis*) leaves were collected as pruning residue and kindly provided by an independent farmer from Espejo (Córdoba, Spain). Before use, they were air-dried under controlled conditions (21 ± 2 °C, 40 ± 5% relative humidity) until constant weight (approx. 10 days) and milled in a home conventional grinder. Sample fraction under 20 mesh size was rejected. Strawberries were purchased from a local supermarket.

The chemical characterization of the samples involved the use of ethanol absolute pure (99.5%, M.W. = 46.07, Panreac, Barcelona, Spain), toluene purissimum (99.5%, M.W. = 92.14, Panreac, Spain), sulfuric acid (95–98%, USP-NF, BP, Ph. Eur., PRS-CODEX, M.W. = 98.08, Panreac, Spain), acetic acid glacial (99.7%, Reag. USP, Ph. Eur., for analysis, ACS, ISO, M.W. = 60.05, Panreac, Spain), sodium chlorite (solution 25% *w*/*w*. for synthesis, M.W. = 90.44, Panreac, Spain), sodium hydroxide (≥97%, pellets EMPLURA^®^, M.W. = 40.00, Merck, Madrid, Spain), 2,2′-Azino-bis(3-ethylbenzothiazoline-6-sulfonic acid) diammonium salt (ABTS, ≥98%, M.W. = 548.68, Sigma-Aldrich, Shanghai, China), potassium persulfate (≥99%, ACS reagent, M.W. = 270.32, Sigma-Aldrich, Darmstadt, Germany), Folin and Ciocalteu′s phenol reagent (2 M with respect to acid, Sigma-Aldrich, Buchs, Switzerland), gallic acid (≥98%, anhydrous for synthesis, M.W. = 170.12, Merck, Darmstadt, Germany), and sodium carbonate (≥99.5%, ACS reagent, M.W. = 105.99, Sigma-Aldrich, Germany).

Culture media were prepared using bacteriological peptone (LP0037B, Oxoid, Hampshire, UK), yeast extract (LP0021B, Oxoid, Dardilly, France), bacteriological agar (Agar No. 1, LP0011B, Oxoid, Madrid, Spain), D(+)-Glucose (Reag. Ph Eur, anhydrous for biochemistry, M.W. = 180.16, Merck, Martillac, France), and sodium chloride (pure, pharma grade, USP, BP, Ph. Eur., JP, M.W. = 58.44, Panreac, Barcelona, Spain).

For composites preparation, carboxymethyl cellulose (sodium salt, average M.W. = 250,000, DS = 0.9, Acros Organics, Illkirch-Graffenstaden, France), gum Arabic (general purpose grade, powder, Fisher Scientific, Loughborough, UK), polyvinylpyrrolidone (M.W. = 360,000, powder, Alfa Aesar, Karlsruhe, Germany), and polyvinyl alcohol (98–99% hydrolyzed, low molecular weight, Alfa Aesar, Germany) were procured.

### 2.2. Bay Leaves Extraction Process

Bay leaf extract (BLE) was obtained following the method of Rincón et al., 2019 [14]. Thus, a conventional Soxhlet apparatus was used to perform an ethanolic extraction of bay leaves, introducing 5 g of sample in cellulose thimbles and adding 200 mL of absolute ethanol, keeping the process up to 5 h. The liquid fraction was then concentrated under vacuum (rotary evaporator Laborota 4000, Heidolph Instruments GmbH&Co, Shwabach, Germany) to around 10 mL, and both the extraction yield and the final concentration were determined. The resulting oily extract was stored for further analysis. Regarding the solid fraction obtained, it was air-dried and subsequently characterized to determine the inorganic matter, extractives, acid-soluble lignin, α-cellulose, and hemicelluloses contents [15]. For this procedure, 6 replicates were made.

### 2.3. Bay Leaves Extract Characterization

The main components in BLE were analyzed on an Agilent Technologies GC–MS Instruments 5977B GC/MSD, Inc., Santa Clara, CA, USA, US equipped with an HP-5 ms Ultra Inert column (30 m × 250 μm × 0.25 μm). GC-MS spectra were obtained using the following conditions: carrier gas, He; flow rate, 1.8 mL/min; split, 1:20; injection volume, 1 μL; injection temperature, 300 °C; oven temperature, 200 °C; the ionization mode used was electronic impact at 70 eV. The identification of the components was achieved by comparing mass spectral fragmentation patterns with those stored in the data bank NIST Standard Reference Database 1Av17 (Data Version NIST v17, Software Version 2.3). The relative content (referred to as the % of the total area) of the main compounds was determined by the integration of peaks according to the method followed by Koudehi et al., 2020 [16].

Total Phenolic Content (TPC) in BLE was determined according to the Folin–Ciocalteu method previously described by García et al., 2017 [15] with slight modifications, thus using gallic acid as the standard compound (calibration curve) and ethanol as solvent. Briefly, 0.5 mL of sample was mixed with 2.5 mL of Folin–Ciocalteu reagent, 5 mL of 0.2 M Na_2_CO_3_, and distilled water up to 50 mL. After 30 min in a thermostatic bath at 40 ± 2 °C, the sample was measured in a Perkin Elmer UV/VIS Lambda 365 spectrophotometer, Inc Shelton, US at 750 nm against a reference (containing 0.5 mL of ethanol). TPC was determined by triplicate and expressed as mg of gallic acid equivalents by g of sample (mg GAE/g).

Antioxidant power (AOP) of BLE was evaluated according to the ABTS (2,2′-Azino-bis(3-ethylbenzothiazoline-6-sulphonic acid)) spectrophotometric assay at 734 nm in a Perkin Elmer UV/VIS Lambda 365 spectrophotometer [15]. Briefly, 7 mM ABTS in 2.45 mM of potassium persulfate was prepared and left overnight in darkness. For the measurement, 0.02 mL of BLE was added to 2 mL of the radical solution in 50% vol. ethanol-water (with an absorbance of 0.7 ± 0.02 at 734 nm). A blank sample (0.02 mL of ethanol) was also measured, both against the radical solution in 50% vol. ethanol-water. *AOP* calculation was done as follows:

$$AOP\left(\%\right)=\frac{A_{blank6}^{734}-A_{BLE6}^{734}}{A_{ABTS0}^{734}}\times 100$$

where *A*^734^*_ABTS0_* is the absorbance of the radical solution at 0 min, and *A*^734^*_blank_*_6_, *A*^734^*_BLE_*_6_ are the absorbances after 6 min room incubation of the blank and BLE samples, respectively.

The antimicrobial activity of the BLE was evaluated against four different microbial strains provided by the Department of Microbiology of the University of Córdoba (i.e., *Escherichia coli*, *Bacillus cereus*, *Candida glabrata*, and *Saccharomyces cerevisiae*). Growth inhibition was determined following three different approaches. In the first one, the method followed by Diba et al., 2011 [17] was adapted. Thus, serial volumetric dilutions of BLE (1/20 to 1/160) were prepared on nutrient broth for bacteria (0.3% yeast extract, 0.5% peptone, 0.5% NaCl, 2% agar) and YPD broth for yeasts (1% yeast extract, 2% peptone, 2% glucose, 2% agar). Then, inoculation was carried out, incubating them at 28 ± 2 °C for *B. cereus*, *C. glabrata*, *S. cerevisiae*, and at 37 ± 2 °C for *E. coli* for a duration of 24 h. After incubation, it was qualitatively determined if there was microbial inhibition through light microscopy (Gram staining for bacteria and simple staining for yeasts). In the second approach, the disk-diffusion method originally introduced by Kirby-Bauer was followed, with minor adjustments made to the procedure described by Șachir et al., 2022 [18]. Briefly, after the inoculation of each culture medium, disks were impregnated with 50 μL of each previously prepared BLE dilution and subsequently incubated under the same conditions as in the first approach. Finally, the third test consisted of the agar-well method. To accomplish this, the procedure outlined by Chau et al., 2022 [19] was followed to assess whether the nature of the disks interfered with the diffusion capacity of BLE in the culture medium. All assays were conducted in triplicate, using culture medium lacking BLE as negative controls. The final aim was to determine the minimum inhibitory concentration (MIC), defined as the lowest concentration of the substance that can inhibit the visible growth of the microbial strain after being incubated for 24 h at its optimal temperature growth. BLE was subsequently categorized based on its MIC by using the classification method described by Aligiannis et al., 2001 [20]. Thus, BLE can be considered a strong inhibitor (MIC up to 0.5 mg/mL), a moderate inhibitor (MIC ranging from 0.6 mg/mL to 1.5 mg/mL), or a weak inhibitor (MIC above 1.6 mg/mL).

### 2.4. Edible Films Preparation and Characterization

Edible films were prepared using the solvent casting method, dissolving 0.5 g of different polymeric matrices (i.e., carboxymethyl cellulose (CMC), polyvinyl pyrrolidone (PVP), polyvinyl alcohol (PVA), and gum Arabic (GA)) in 20 mL of solvent containing 0, 5, 10 and 15% wt. of BLE respect to polymer. Water was used as the solvent for CMC, PVA, and GA films, whereas ethanol was chosen for dissolving PVP [21,22]. To reduce GA composite fragility, glycerol (10% wt. respect to polymer) was used as a plasticizer. Subsequently, casting was conducted for 72 h at 21 ± 1 °C, performing a final vacuum drying (Vacuo-Temp JPSELECTA) at 50 ± 2 °C for total solvent removal. All the films were prepared in triplicate and individually stored until their characterization, placing them in a conditioned room (25 ± 1 °C, 50% RH) for 72 h before characterization to standardize the starting conditions.

The water vapor permeability (WVP) of films was determined according to the ASTM E96/E96M-10 standard [21]. Shortly, a film square of 4 cm^2^ was cut from each composite and attached to an aluminium adhesive tape. Then, the system was arranged in plastic jars, whose lids were previously perforated with ≈10 mm diameter holes, and finally, a desiccant material (CaCl_2_) was added. Subsequently, jars were placed in a controlled chamber (25 ± 1 °C, 50% RH), and their weight was recorded at 0, 1, 2, 3, 4, 5, 6, 7, and 24 h. Three replicates were measured for each sample. Thus, *WVP* (g·Pa^−1^·m^−1^·h^−1^) was calculated as:

$$WVP=\frac{G\cdot e}{t\cdot A\cdot P\cdot H}$$

where *G* (g) is the weight gain slope in a time *t* (s) through an area *A* (m^2^) of a film with a thickness *e* (m) tested at a pressure *P* (Pa) and specific humidity *H* (expressed as per one). *WVP* measurements were determined by triplicate on each composite.

Furthermore, the light transmission was investigated in a Perkin Elmer UV/VIS Lambda 365 spectrophotometer in the 200–700 nm range to evaluate the opacity and UV-light protective capacity of the prepared films, according to the method of Rincón et al., 2019 [14]. Thus, specific transparency (*ST*, %/mm) and UV-light blocking (*UV_block_*, %) were calculated as follows:

$$ST=\frac{\log T_{600}}{e}\;UV_{block}=100\cdot \left(1-\frac{T_{280}}{T_{660}}\right)$$

where *T*_600_, *T*_660_, and *T*_280_ are the transmittances (%) recorded at 600, 660, and 280 nm, respectively, and *e* (mm) represents the thickness of the tested films. These measurements were performed in triplicate for each sample.

### 2.5. Fruit Protection Tests

The application of edible coatings was performed on similar strawberries (shape, weight, and ripening stage). Each specimen was immersed in its respective emulsion (a concentration of 2.5% wt. of dissolved pure matrix or composite, i.e., of dissolved polymer and with either 0% wt. or 15% wt. of BLE). In the case of GA-based samples, a 10% wt. of glycerol was also considered in the final 2.5% wt. concentration of the prepared emulsion. The immersion process lasted for 10 s, followed by drying on Petri dishes and storage under controlled conditions (21 ± 2 °C and 40 ± 5% RH). Uncoated fruits (UF) were used as controls for comparison purposes. Then, mass loss (%) was determined through gravimetry on days 1, 2, 5, and 6, while the appearance of the samples was evaluated through photographs taken for 12 days, carrying out in both cases three replicates for each specimen.

### 2.6. Statistical Analysis

Data are shown as means ± standard deviation. To carry out the statistical analysis, IBM SPSS software version 25 for Windows was used, performing one-way ANOVA tests to determine the differences between the groups for multiple comparisons, while Tukey post-hoc tests were used to identify which groups differed. The level of significance was fixed at 5%.

## 3. Results and Discussion

### 3.1. BLE Extraction and Characterization

**Figure 1:**
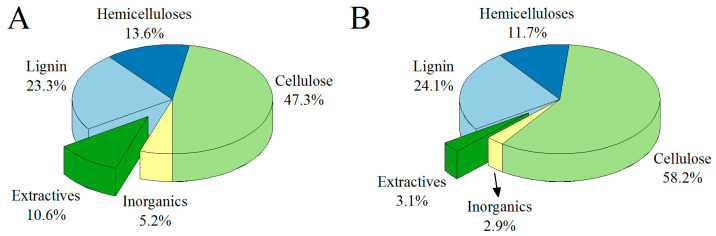
Composition (% wt. basis) of bay leaves fibers before (**A**) and after (**B**) ethanolic extraction.

The ethanolic extraction of the raw material resulted in the removal of 22.3 ± 1.2% wt. of the starting components, thus producing an oily extract with a concentration of 0.104 g/mL of total dissolved solids. The obtained extraction yield was observed to be lower than previously reported for similar techniques, solvents, and raw materials [14]. This discrepancy can be attributed to various factors that can influence this value, including the climatic conditions of the collection area and the age of the plant, among others [23]. However, it is noteworthy that our extraction yield was higher when compared to other conventional extraction techniques used on BLE extraction, such as the heat-reflux method (10.34 ± 1.58%) or the microwave-assisted extraction method (10.50 ± 0.57%) [24]. This comparison highlights the substantial variability in extraction yields depending on the chosen extraction method, underscoring its crucial role in achieving optimal extraction efficiency. As shown in Figure 1, most of the extractable and inorganic matter was removed after the ethanolic extraction (around 70 and 44% of the initial content, respectively), in addition to the partial dissolution of hemicelluloses (approximately 14% of the initial content).

The identification and relative quantification (% relative area, RA%) of the different bioactive compounds in BLE are shown in Table 1, according to the results of the performed GC/MS analysis. In general, the amount and type of bioactive compounds present in EOs depend on many factors such as the extraction method used, the geographical origin of the raw material, whether it is wild or cultivated plants, etc. In the present work, BLE had a significant presence of monoterpenoid compounds (methyl-4,6-O-benzylidene-hexopyranose), also appearing monoterpenoids (1,8-cineole, isopulegol, and α-terpineol), phenylpropanoids (methyl eugenol and elemicin), and sesquiterpenoid compounds (β-acorenol, β-caryophyllene, and cedrandiol), thus coinciding with the composition reported by other authors [25]. Moreover, it has been reported that the presence of methyl-4,6-O-benzylidene-hexopyranose, isopulegol, α-terpineol, methyl eugenol, elemicin, β-acorenol, β-caryophyllene, and cedrandiol can induce synergistic microbiocidal action against most common pathogens that threaten food quality and safety such as *Pseudomonas* spp., *Leuconostoc* spp., *Saccharomyces* spp., *Penicillium* spp., and *Fusarium* spp., among others [26].

In terms of the TPC of BLE, the value obtained (118 ± 4 mg GAE/g) aligns well with the findings of previous research conducted on *L. nobilis* leaves. For instance, Dhifi et al., 2018 [34] reported a TPC of 174.1 ± 11.6 mg GAE/g for methanolic extracts obtained by stirring. In fact, this value is deeply influenced by both the polarity of the solvent used and the extraction method employed, playing a key role in determining the potential bioactivities associated with BLE and its applicability. The significant presence of phenolic compounds in our BLE extract suggests its potential as a sustainable additive in food coating, given their well-known properties in promoting the preservation and protection of food products.

Indeed, phenolic compounds are often correlated to the AOP due to their capability to act as electron donors in a free radical reaction [35]. In the present research, ABTS radical reduction power of 75.06% was obtained. This value was 1.6 times higher than that obtained by Muñiz-Márquez et al., 2014 [36], although they carried out a heat-reflux extraction, which also supports the conclusions reached above. The obtained results indicate a great antioxidant capacity of BLE, promising for its application in food protection against oxygen free radicals.

**Figure 2:**
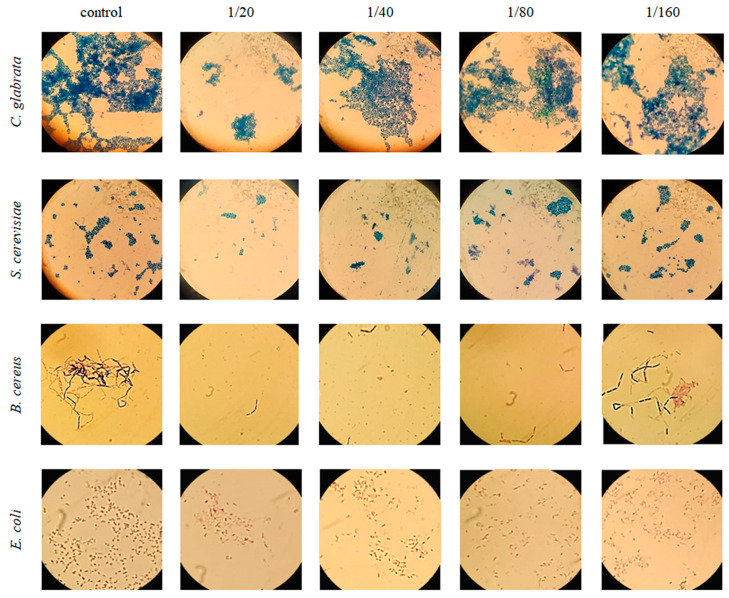
Effect of BLE concentration (left to right: dilution increasing) on the growth of the tested microorganisms (top to bottom: *C. glabrata*, *S. cerevisiae*, *B. cereus*, *E. coli*).

According to the microbial inhibition tests (Figure 2), the microscopy analysis of serial dilutions of BLE showed the tolerance of *E. coli* against the ethanolic extract, a behavior corroborated after observing the growth of the bacteria in the solid medium, where no inhibition halos were obtained in any of the two proposed tests. These results match the information cited in the literature [37], so we can rely on the nature of the outer membrane to explain them, as it probably limits the passage of some bioactive compounds present in BLE. Referring to *B. cereus*, a biocidal power of BLE was observed in all dilutions made (MIC = 0.65 mg/mL, moderate inhibitor [20]), observing both a decrease in the number of bacilli and a decrease in forming associations, thus agreeing with the results obtained by Btissam et al., 2018 [38]. This behavior was corroborated through the Kirby-Bauer and agar-well assays, observing inhibition halos in all the dilutions of the ethanolic extract tested. In this way, the results obtained agree with authors who state that Gram-positive bacteria are more sensitive than Gram-negative bacteria to aromatic plants EOs [38]. Finally, considering the results obtained with the two yeasts tested, it was found that BLE hardly caused growth inhibition in any of them. However, other studies have shown inhibition in the growth of *S. cerevisiae* with a considerably lower concentration (0.02%) of laurel EO [39], while other tests with *C. glabrata* have shown that *L. nobilis* EOs are able to inhibit its growth [40]. Nevertheless, in both investigations, they extracted aqueous extracts instead of ethanolic extracts from the plant, so in the absence of previous studies testing ethanolic extracts, our results confirm the presence of levuricidal and/or levuristatic compounds in the water-soluble fraction of bay leaves. Ultimately, these results encourage the search for other approaches to enhance the antimicrobial power of the chemical compounds present in *L. nobilis* EO by modifying the technique, time, or solvent used in the extraction to test its effectiveness against other food spoilage microorganisms.

### 3.2. Properties of BLE Containing Edible Films

Depending on the food to be protected, a film must allow moisture transfer from the atmosphere to a greater or lesser extent. Considering our results for WVP (Table 2), the incorporation of BLE in CMC-based biocomposites caused a decrease in their permeabilities (between 41% and 48%, referred to the value observed for CMC0), becoming more significant as extract content increased. Regarding GA-based composites, BLE determined a reduction in the WVP of Arabic gum between 40% and 75%. Interestingly, a percolation threshold seemed to appear at GA5, thus determining an increase in its value, although from GA10 onwards the WVP again decreased considerably. The appearance of percolation thresholds in composites is generally due to a heterogeneous distribution of components on the polymer matrix. In this sense, the extremely randomized structure of GA leads to weaker interactions with BLE components. In addition to this, most of its interactions are through the -OH group, much weaker than in the case of CMC, whose hydroxyl group is more polarized by the presence of adjacent carbonyl groups. The abrupt change in WVP observed for GA10 and the great variability in its standard deviation suggest the probable presence of pores or a worse dispersion of the BLE during the preparation of these samples. For PVP-based films, BLE incorporation linearly reduced the water vapor transference through the biocomposite (22%, 43%, and 65% less when 5%, 10%, and 15% of extract was added, respectively). However, these differences were not significant (*p* > 0.05) as indicated by the statistical analysis, probably due to the great variability among the replicates. Lastly, PVA-based biocomposites were those that allowed the transfer of water vapor from the atmosphere to a lesser extent. In them, an apparent reduction in the WVP value (between 47% and 74%) was also observed when BLE was incorporated. Furthermore, a percolation threshold seemed to appear in PVA10, determining a slight increase in the property. In essence, the occupation of free spaces by BLE generated more difficult pathways for water molecules, subsequently reducing WVP [21]. Thus, an increase in the water vapor barrier is related to the content of solids and polyphenols, components that interact with the polymer and make the film matrix more compact.

Given the effect of opacity on the consumer acceptability of a product, it is crucial that the prepared formulations have the highest possible ST. In our case, the addition of BLE determined significant differences in the ST of CMC, GA, and PVA-based films, determining its increase or decrease depending on the concentration and type of polymer matrix, although there was a generalized maintenance of transparency in the presence of all BLE concentrations tested, as shown in Table 2. The clearest example of this fact was found in the PVP-based composites, where the differences found with respect to the control were not statistically significant. Thus, considering the values obtained for ST, the most transparent films were those formed using PVA as the polymer matrix (T660 nm > 90%), followed by CMC, PVP, and GA, respectively. The maintenance of transparency in our composites despite the addition of BLE is in agreement with Oun et al., 2022 [41], who state that the type of polymer used, the concentration of the additive, its dispersibility, and compatibility with the polymer matrix can determine an increase or reduction in the ST, indistinctly, as has been demonstrated in other works to date, although a generalized non-modification of the property is related to a good dispersion of the additive in the polymeric matrix. On the other hand, the reduction in the ST obtained in some of the analyzed biocomposites containing BLE is justified by the presence of a lipid phase capable of scattering light, as reported by other authors [42].

Furthermore, due to long hours of customer service in supermarkets, multiple foods are continuously exposed to fluorescent light, leading to vitamins, pigments, amino acids, and fats photodegradation [43]. Considering our results for UV_block_ improvement (Table 2), the addition of BLE significantly improved the property, achieving total blocking (>99.90%) in all films, even at 5% wt. The observed increase in GA-based films was particularly remarkable, exhibiting a substantial rise from 8.25% to 99.94%. In contrast, the improvement was less evident in PVA-based composites, as in the absence of BLE they were able to block UV radiation up to 48%. According to previous research, the UV light absorption capacity of ethanolic extracts of *L. nobilis* has been specifically associated with the presence of chromophore groups in them [44]. Similarly, Moustafa et al., 2023 [45] stated that this considerable enhancement of UV blocking ability is generally caused by aromatic rings present in essential oils, making it a broad natural protector against UV radiation across the entire UV-visible spectrum.

### 3.3. Protection/Coating of Fresh Fruit

Analyzing the results obtained for mass loss (Table 3), it can be established that most of the impregnation treatments (both including or not BLE) significantly favored the maintenance of the property over time with respect to the control (46.72 ± 1.96% on day 6), an effect accentuated after 5 and 6 days of testing. Indeed, the only exception was the treatment with GA0, whose application determined a mass loss even slightly higher than the trials in the absence of coating (48.01 ± 1.64% on day 6). This behavior can be explained due to the nature of the polymer, which provides an extra carbon source and determines a greater susceptibility to being attacked by microorganisms that cause fruit spoilage such as *Alicyclobacillus acidoterrestris* or *Byssochlamys nivea* [46]. On the other hand, strawberries impregnated with both CMC and PVP presented a mass loss similar to each other (32.17 ± 2.34% and 29.13 ± 0.36%, respectively, after 6 days), being in any case significantly lower than that obtained for UF. Finally, strawberries impregnated with the PVA15 biocomposite showed the least variation in mass over time (22.13 ± 0.77% on day 6). These results agree with those obtained for WVP, confirming that the most permeable coating material is the one that further determines the greatest mass loss of strawberries, while the least permeable material is the one that has managed to maintain the mass of the strawberries tested to a greater extent. In this regard, a Pearson correlation coefficient of 0.85 was found, indicating a positive and direct relationship between these two parameters. Furthermore, the results obtained in relation to the addition of BLE agree with the conclusions reached after its characterization, since the long-term mass loss value is lower in the biocomposites containing it with respect to those lacking BLE, except in the case of impregnation with CMC, where the values obtained were similar to each other (≈30%).

**Figure 3:**
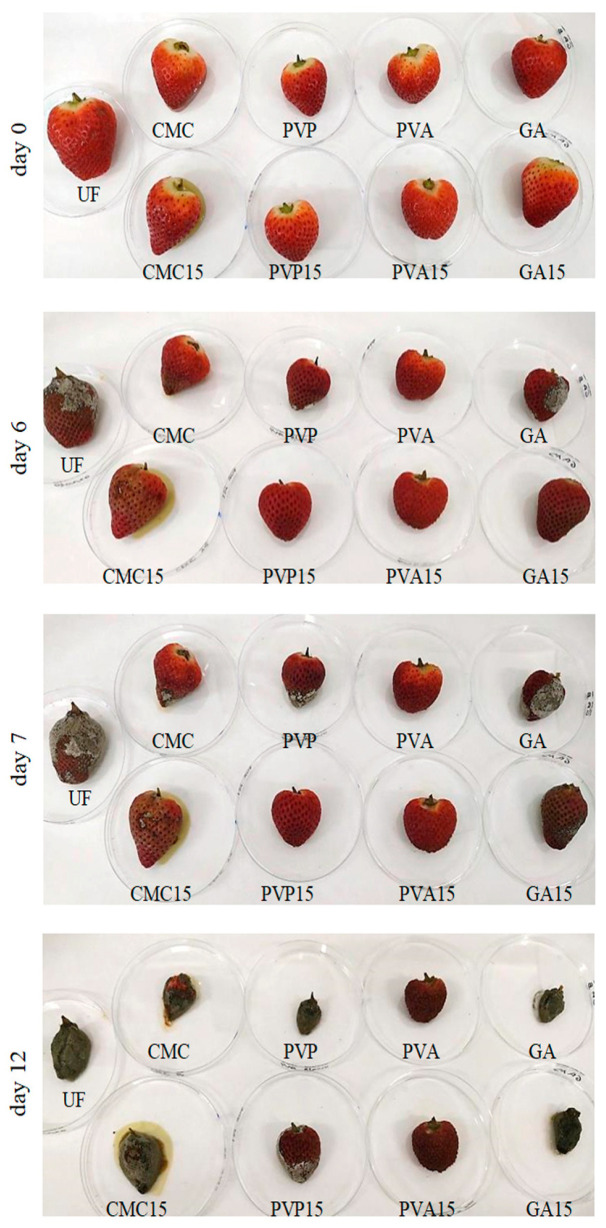
Biodegradation process under controlled conditions (21 ± 2 °C, 40 ± 5% RH) of both uncoated and coated fruits containing 15% wt. of BLE.

The results obtained for mass loss agree with the appearance observed in each of the specimens (Figure 3). Indeed, the pattern of biodegradation over time showed that strawberries impregnated with any biocomposite (except GA0) presented a much better appearance than UF, thus demonstrating the suitability of their application. In particular, the best-preserved ones both in the short and long term were those coated with PVA (regardless of the presence of BLE), followed by those coated with PVP15. Furthermore, the photographs taken showed that the presence of BLE in composites resulted in better maintenance of the mass of the specimens while preserving, as was also indicated by the mass loss values. Finally, as the values obtained for ST showed, it could be observed that the impregnation treatments did not greatly affect the appearance (color) of the strawberries despite containing BLE, which would not pose a problem in terms of potential consumer acceptability on the market.

## 4. Conclusions

In this work, a set of edible strawberry coatings based on the composites formed by different polymeric matrices and the ethanolic fraction of *L. nobilis* leaves (BLE) were designed. The identification of several bioactive chemical compounds in BLE justified its moderate inhibition against *B. cereus* during in vitro tests. Furthermore, the addition of this extract into polymeric matrices allowed to block almost completely the UV radiation (>99.9%) while maintaining the transparency of the composites. Also, it determined an improvement in fruit moisture protection during the coating assays (overall decrease in WVP between 47% and 75% compared to the pure matrix). This maintained a better appearance of coated strawberries over time in CMC15, GA15, PVP15, and PVA15. Overall, it can be concluded that the prepared composites are suitable for the protection of fresh fruit. It is expected to continue research on this subject by evaluating other properties such as organoleptic features (odor, flavor...), additional barrier properties (O_2_ and CO_2_ permeabilities...), thermomechanical characteristics (DMA analysis, burst test...), as well as their applicability in other food products.

## Figures and Tables

**Table 1:** Identification and quantification of bioactive compounds present in BLE sample (RT, retention time; CD, carbohydrate derivative; ME, monoterpenoid ether; MA, monoterpenoid alcohol; Ph, phenylpropanoid; S, sesquiterpenoid; SA, sesquiterpenoid alcohol; relative area, RA %).

RT (min)	Compound	Type	Structure	RA * (%)	Bioactivity	Refs.
1.07	Methyl-4,6-O-benzylidene-hexopyranose	CD	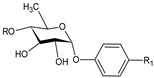	8.08	Antimicrobial	[27]
1.17	1,8-cineole	ME	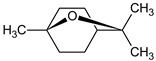	17.65	Insecticidal	[28]
1.28	Isopulegol	MA	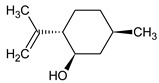	5.17	Antibacterial	[29]
1.45	α-terpineol	MA	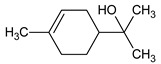	4.98	Antibacterial	[30]
1.53	Methyleugenol	Ph	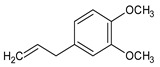	5.44	Antibacterial	[30]
1.88	Elemicin	Ph	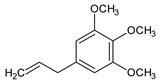	2.87	Antibacterial	[31]
1.65	β-acorenol	SA	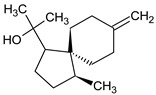	3.38	Antimicrobial	[32]
2.17	β-caryophyllene	S	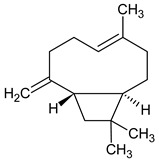	1.32	Antimicrobial	[30]
2.45	Cedrandiol	SA	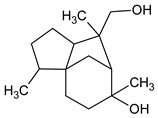	0.89	Antimicrobial	[33]

* The relative area (RA) on chromatograms obtained from the composition analysis was determined in triplicate, consistently resulting in a standard deviation of less than 5% for its value.

**Table 2:** Barrier and optical properties of prepared composites using different matrices and %wt. of BLE.

Composite	WVP (g·Pa^−1^·m^−1^·h^−1^·10^−6^)	ST (%/mm)	UV_block_ (%)
CMC0	2.89 ± 0.19	29.42 ± 0.01	37.22 ± 2.63
CMC5	1.68 ± 0.39	28.05 ± 0.03 (*)	99.94 ± 0.00 (*)
CMC10	1.58 ± 0.30 (*)	30.46 ± 0.00 (*)	99.93 ± 0.00 (*)
CMC15	1.52 ± 0.32 (*)	26.26 ± 0.01 (*)	99.91 ± 0.00 (*)
GA0	10.53 ± 1.39	23.03 ± 0.01	8.25 ± 0.45
GA5	2.64 ± 1.47 (*)	28.21 ± 0.03 (*)	99.94 ± 0.00 (*)
GA10	6.28 ± 2.83	22.38 ± 0.03 (*)	99.93 ± 0.00 (*)
GA15	2.75 ± 1.63	31.55 ± 0.02 (*)	99.92 ± 0.00 (*)
PVP0	5.55 ± 1.05	27.01 ± 0.33	17.63 ± 3.07
PVP5	4.31 ± 1.16	25.28 ± 0.26	99.94± 0.00 (*)
PVP10	3.14 ± 1.21	27.26 ± 0.09	99.93 ± 0.00 (*)
PVP15	1.96 ± 0.71	28.99 ± 1.03	99.92 ± 0.00 (*)
PVA0	1.64 ± 0.30	39.12 ± 0.01	47.99 ± 5.95
PVA5	0.86 ± 0.41	41.38 ± 0.01 (*)	99.94 ± 0.00 (*)
PVA10	0.43 ± 0.20	37.61 ± 0.01 (*)	99.92 ± 0.00 (*)
PVA15	0.58 ± 0.26	36.08 ± 0.00 (*)	99.92 ± 0.00 (*)

Statistical analyses were performed over each set of composites, comparing the properties of each polymeric matrix with its BLE containing set of samples. (*) denotes significant differences between control and composites using Tukey post-hoc tests (*p* < 0.05).

**Table 3:** Mass loss (%) over time in uncoated (UF), coated lacking BLE (0% wt.) and coated containing BLE (15% wt.) strawberries.

Coating	Mass Loss (%)			
	Day 1	Day 2	Day 5	Day 6
UF	5.22 ± 0.37	11.23 ± 0.97	34.54 ± 1.78	46.72 ± 1.96
CMC0	3.64 ± 0.43 (*)	10.45 ± 0.38	23.38 ± 1.75 (*)	32.17 ± 2.34 (*)
CMC15	3.32 ± 0.23	9.42 ± 0.18	22.11 ± 0.29 (*)	27.82 ± 0.41 (*)
GA0	5.02 ± 0.40	10.04 ± 0.42	36.89 ± 1.89	48.01 ± 1.64
GA15	3.48 ± 0.54 (*)	8.07 ± 0.31 (*)	27.23 ± 1.20 (*)	37.21 ± 0.91 (*)
PVP0	4.67 ± 0.16	10.76 ± 0.51	25.30 ± 0.67 (*)	29.13 ± 0.36 (*)
PVP15	5.65 ± 0.47	11.12 ± 0.66	26.60 ± 0.31 (*)	30.39 ± 0.17 (*)
PVA0	2.52 ± 0.56 (*)	6.05 ± 0.88 (*)	18.92 ± 0.54 (*)	26.69 ± 0.48 (*)
PVA15	3.87 ± 0.22	8.25 ± 0.05 (*)	19.60 ± 0.19 (*)	22.13 ± 0.77 (*)

Statistical analysis was performed comparing the mass loss of uncoated fruit with those coated with polymer/composite containing BLE. (*) denotes significant differences between the behavior of UF and coated strawberries using Tukey post-hoc tests (*p* < 0.05).

## Data Availability

The data used to support the findings of this study can be made available by contacting the corresponding author.
